# A fully automated classification of third molar development stages using deep learning

**DOI:** 10.1038/s41598-024-63744-y

**Published:** 2024-06-07

**Authors:** Omid Halimi Milani, Salih Furkan Atici, Veerasathpurush Allareddy, Vinitha Ramachandran, Rashid Ansari, Ahmet Enis Cetin, Mohammed H. Elnagar

**Affiliations:** 1 Department of Electrical and Computer Engineering, University of Illinois Chicago, Chicago, IL USA; 2 Department of Orthodontics (M/C 841), College of Dentistry, University of Illinois Chicago, 801 S. Paulina Street, RM 131, Chicago, IL 60612-7211 USA

**Keywords:** Dentistry, Medical imaging

## Abstract

Accurate classification of tooth development stages from orthopantomograms (OPG) is crucial for dental diagnosis, treatment planning, age assessment, and forensic applications. This study aims to develop an automated method for classifying third molar development stages using OPGs. Initially, our data consisted of 3422 OPG images, each classified and curated by expert evaluators. The dataset includes images from both Q3 (lower jaw left side) and Q4 (lower right side) regions extracted from panoramic images, resulting in a total of 6624 images for analysis. Following data collection, the methodology employs region of interest extraction, pre-filtering, and extensive data augmentation techniques to enhance classification accuracy. The deep neural network model, including architectures such as EfficientNet, EfficientNetV2, MobileNet Large, MobileNet Small, ResNet18, and ShuffleNet, is optimized for this task. Our findings indicate that EfficientNet achieved the highest classification accuracy at 83.7%. Other architectures achieved accuracies ranging from 71.57 to 82.03%. The variation in performance across architectures highlights the influence of model complexity and task-specific features on classification accuracy. This research introduces a novel machine learning model designed to accurately estimate the development stages of lower wisdom teeth in OPG images, contributing to the fields of dental diagnostics and treatment planning.

## Introduction

Determination of tooth development stages or dental age holds profound significance within the dental and medical field as well as forensic practice^1^. Dental age refers to the development and condition of an individual’s teeth, while chronological age is simply the number of years a person has lived^2^. Typically, dental age can provide insights into an individual’s overall growth and development. It is important to note that dental age can vary among persons of the same chronological age. Factors such as genetics and overall health can influence dental development. Some individuals may have teeth that mature faster or slower than their peers, resulting in variations between dental age and chronological age^3,4^. This variability underscores the importance of accurate assessment methods, such as the Demirjian classification system, which offers a standardized approach to evaluating dental maturity.

The Demirjian classification system is widely used to assess dental age and tooth development stages using orthopantomo- grams (OPGs) also known as panoramic radiographs. This system categorizes tooth development into eight stages, from A to H, based on the appearance and mineralization of the third molar ^5,6^. The Demirjian method has been extensively studied and validated. It has shown good accuracy in estimating dental age in various populations^7–9^. However, it is important to note that the reliability of any classification method heavily depends on the experience and expertise of the dentist analyzing the OPGs. Errors and inconsistencies can occur when different dentists interpret the same OPGs, highlighting the need for a standardized and automated approach. These errors can impact treatment decisions and patient outcomes. In addition, when it is applied in medicolegal and forensic contexts, the limitation is its reliance on experts for analysis. The integration of AI and ML technologies presents a promising solution to these challenges by potentially automating the analysis process, thereby enhancing accuracy and consistency across dental assessments. This shift towards technological solutions marks a crucial development in our approach to dental and forensic analysis, bridging traditional methods with the cutting-edge capabilities of AI. This can result in delays and potentially impede the swift resolution of legal cases. Addressing these challenges, the advent of AI and ML technologies heralds a new era in dental and forensic analysis, offering a pathway to overcome the limitations of manual assessments. The development of an automated and reliable tool for dental age and tooth development assessment would be highly advantageous, as it would reduce dependence on human interpretation and ensure more efficient processes, particularly in legal and forensic scenarios^10–12^.

Despite the proven utility of the Demirjian system and other traditional methods, there remains a significant gap in the literature regarding the comprehensive automation of such assessments using AI. This gap highlights an urgent need for innovative research focused on the application of AI in dental age assessment. Addressing this gap, our study presents a novel contribution by developing a sophisticated deep learning model that significantly enhances the accuracy and reliability of dental age assessment. The application of Artificial Intelligence (AI) and machine learning (ML) techniques in the realm of medical imaging for health care and forensic applications is undergoing rapid evolution. The prospect of a fully automated diagnostic or analysis approach has garnered attention for its potential to diminish human error, along with the associated time and effort required for the task. While our study concentrates on the automated classification of lower third molar development stages for dental age assessment, we recognize the potential implications of our findings in forensic dentistry. This acknowledgment paves the way for our exploration of deep learning techniques, especially CNNs, to not only advance dental age assessment but also to explore their broader utility in forensic applications. The application of CNNs, as demonstrated in our research, could extend to forensic applications, facilitating the identification process in challenging scenarios through detailed dental analysis ^13^. In recent times, a sophisticated ML technique known as “deep learning (DL)” has emerged as the preeminent tool for addressing image pattern recognition and classification issues, finding widespread utilization in the medical domain^14–18^.

An automated, accurate AI system for dental age assessment based on the Demirjian classification system would address the limitations of human interpretation, reduce potential errors and inconsistencies, expedite the analysis process, and broaden the accessibility of this method. It has the potential to greatly improve the efficiency and accuracy of dental age assessment, particularly in medicolegal and forensic contexts^19^.

Against this backdrop, our research objectives are twofold: first, to establish an automated system for the classification of lower third molar development stages using CNNs; and second, to evaluate the efficacy of this system in improving dental age assessment practices. We hypothesize that our AI-driven approach will demonstrate superior accuracy and reliability compared to traditional methods, offering a significant advancement in both dental and forensic fields. There have been few endeavors in the literature to devise an automated technique for the classification of lower third molar development stages ^20^. De Tobel et al. conducted a pilot study using a relatively small dataset. The authors compared the outcomes of their automated technique with the manual staging carried out by experienced dentists and reported a mean accuracy of 0.51^20^. Although the pilot study yielded promising results, they were not accurate enough for clinical application. Therefore, further research and validation are imperative to establish the reliability and accuracy of the automated technique on a broader scale.

To address the challenges of scalability and robustness identified in prior research, the data used in our study encompasses a diverse demographic, including individuals of different ages and races, ensuring a comprehensive analysis. Our sample size was significantly expanded to include both male and female subjects across various developmental stages. We advanced be- yond traditional approaches by not using a standard pre-trained neural network to classify the developmental stages of lower third molars from orthopantomograms (OPGs). Instead, our approach involves a network that merges trainable filters with elements of a pre-trained network, significantly enhancing the traditional use of pre-trained models. Our model introduces a novel integration of transfer learning with directional high-pass filters and focal loss, which collectively enhance performance. By leveraging transfer learning, we repurpose existing models for related tasks, thereby improving both adaptability and effi- ciency. The addition of directional filters aids in emphasizing critical features and patterns, optimizing the model’s focus for new challenges. Furthermore, we incorporate focal loss to better address class imbalances, prioritizing learning from difficult examples to improve model accuracy. This efficient adaptation of pre-trained models to new tasks leverages the strengths of each component, resulting in superior outcomes.

Therefore, this study aims to leverage AI technology to establish an automated and highly efficient system for detecting and classifying lower third-molar development stages. Our research focuses on the creation of a fully automated deep-learning model for assessing lower third molar mineralization stages. To achieve this, we intend to accumulate and analyze an extensive dataset comprising dental images. For the implementation of deep learning algorithms, particularly convolutional neural networks (CNNs), we will employ these models to extract essential features from dental images to use in the determination of the development of lower third molar.

The key contributions of this paper are as follows:

- A novel Deep Neural Network (DNN) algorithm specifically tailored to this application is used to classify the lower third molar stages.
- The DNN algorithm utilizes preprocessing filters to improve the accuracy of the classification. The weights of the convolutional preprocessing filters are initialized as edge detectors and image smoothers. High and low-frequency components of the panaromical images are highlighted prior to the DNN which leads to a better final result. High-pass filters essentially accentuate the edges of the molar tooth images and low-pass filters produce smoothed images reducing the noise in x-ray images. The strategic application of these preprocessing filters is rooted in their ability to refine the input data for our neural network, enhancing the model’s precision in classifying the development stages of third molars.
- The initial weights of the preprocessing filters are further optimized during the neural network training.
- The proposed model is an entirely automated system capable of autonomously processing images and generating classification results without any manual intervention.

## Materials and methods

In this section, we discuss the data set preparation in this study and introduce the neural network model used for data analysis, while proposing a comprehensive automated methodology that combines Region of Interest (ROI) extraction, carefully selected pre-filtering, and extensive data augmentation techniques. We provide quantitative measures utilized to verify the validity and performance of the study and emphasize key image attributes to improve classification accuracy significantly. Validation metrics and our strategy utilizing a variety of deep neural network architectures, including EfficientNet, EfficientNetV2, MobileNet Large and Small, ResNet18, and ShuffleNet, all optimized for this specific task.

### Preparing the data set

In this research, we conducted a retrospective study utilizing archived data. All data used in the study was deidentified to ensure confidentiality. The University of Illinois Chicago’s Office for the Protection of Research Subjects (OPRS) Institutional Review Board (IRB) granted an exemption from the requirement for informed consent, providing IRB-exempt status to the study with the assigned Study ID: (2021-0480), all methods were performed by the relevant guidelines and regulations of the University of Illinois Chicago’s OPRS and IRP. We performed a comprehensive analysis of Orthopantomograms (OPG’s) and accurately classified them based on the different stages of tooth development using the Demirijian tooth development stages as a reference. Our approach involved meticulous examination of various images to ensure precise classification^6^. To conduct this study, two skilled practitioners meticulously reviewed more than 15,000 orthopantomograms (OPGs) from the patient records database of the University of Illinois, Chicago, College of Dentistry clinics, and implemented a rigorous pre-filtering process. The sample exclusion criteria comprised poor image quality and the lack of essential demographic information. Furthermore, patients with dental or craniofacial anomalies, as well as those affected by syndromes impacting dental or craniofacial structures, were excluded from the study. Additionally, individuals with a history of head and neck trauma or prior head and neck surgery were also excluded. The established protocols and careful handling of the data collection process were designed to ensure its reliability and validity. The OPGs were labeled as the follows: (1) Missing lower 3rd molar. (2) Empty Follicle and the Demirjian Stages are as follows: (3) Stage A: Cusp tips are mineralized but have not yet coalesced. (4) Stage B: Mineralized cusps are united so the mature coronal morphology is well-defined. (5) Stage C: The crown is about half formed; the pulp chamber is evident and dentinal deposition is occurring. (6) Stage D: Crown formation is complete to the dentinoenamel junction. The pulp chamber has a trapezoidal form. (7) Stage E: Formation of the inter-radicular bifurcation has begun. Root length is less than the crown length. (8) Stage F: Root length is at least as great as crown length. Roots have funnel-shaped endings. (9) Stage G: Root walls are parallel, but apices remain open. (10) Stage H: Apical ends of the roots are completely closed, and the periodontal membrane has a uniform width around the root. The principal evaluator conducted a second round of classification two weeks later to assess intra-examiner reproducibility for the Demirjian classification stages, employing weighted kappa (wk) as a measure. The intra-examiner agreement demonstrated near perfection (wk = 0.92). Additionally, a separate evaluator repeated the classification process, revealing strong inter-examiner agreement (wk = 0.90). Both the evaluators are experts, clinician-scientists.

**Figure 1:**
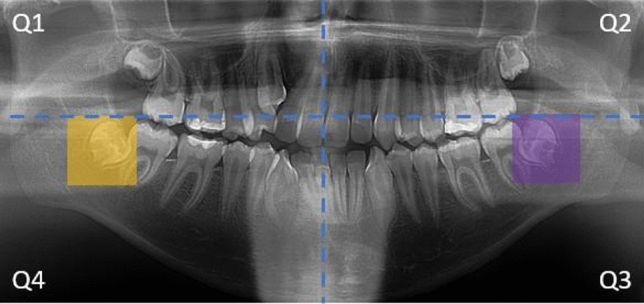
A panoramic X-ray image. Q3 and Q4 wisdom teeth are highlighted.

In this research, we focus on classifying wisdom teeth in panoramic images, specifically using the third and fourth quadrants (Q3 and Q4). Since the region of interest (RoI) is localized around the wisdom teeth, utilizing the entire panoramic image is unnecessary for image classification. Therefore, we aim to extract the RoI through image cropping. Figure 1 shows a panoramic X-ray image sample from our data set. We draw two rectangles over two wisdom teeth located in the third and fourth quadrants. As depicted in Fig. 1, the wisdom teeth are accurately highlighted, cropped, and resized to a standardized dimension of 192 × 192.

**Figure 2:**
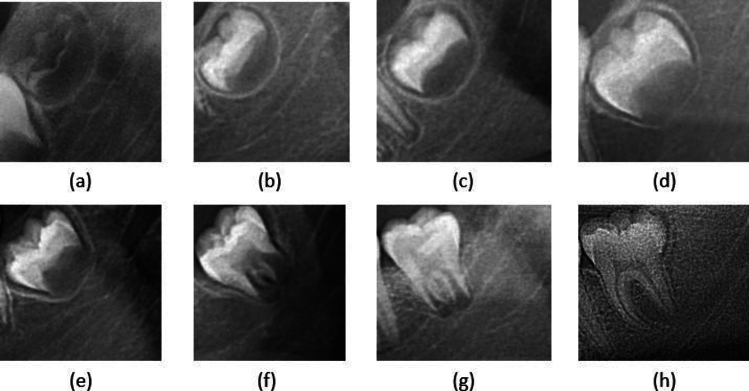
Demirjian stages. Images (**a**–**h**) correspond to stages 3, 4, 5, 6, 7, 8, 9, and 10, respectively.

The final training dataset consists of 3422 OPG, each classified and curated by the primary evaluators. This data set includes both Q3 and Q4 regions extracted from panoramic images, resulting in a collection of 6624 images available for analysis. Among these images, we observe distinct distribution patterns, with 674, 734, 1160, 1062, 870, 593, 410, and 1121 cropped images corresponding to Demirjian stages 3, 4, 5, 6, 7, 8, 9, and 10, respectively. Demirjian stages are illustrated in Fig. 2. We present cropped and resized ROI of wisdom teeth taken from each class.

One important consideration regarding the data set is the potential imbalance in sample distribution across different classes. To address this issue, two effective strategies can be employed: data augmentation and weighted data sampling. Data augmentation is a widely used technique that helps to overcome model overfitting by generating additional samples during each iteration of model training. In this study, we apply various augmentation methods such as rotation, auto contrast, and translation to diversify the data^21^. These techniques introduce subtle variations to the existing samples, effectively expanding the data set. Furthermore, to ensure a balanced representation of each class during both training and testing, we implement a weighted data sampler^22^. The weighted data sampler assigns higher probabilities to underrepresented classes, which ensures that the model encounters a similar number of images from each class during the training process. This approach helps prevent bias towards classes with larger sample sizes and allows the model to learn from the entire data set more effectively, ultimately leading to improved classification performance. By utilizing data augmentation and a weighted data sampler, our model becomes more robust and better equipped to handle the imbalanced data distribution inherent in medical imaging tasks.

The data partitioning for training and testing is carefully performed, ensuring a robust evaluation of the proposed approach. Specifically, 5300 images are allocated for training the model, while the remaining 1324 images are reserved for testing and validation.

### Deep neural networks used in this study

The Convolutional Neural Network (CNN) is one of the most efficient and commonly used trainable models for image classification tasks. It has proven to be highly effective in categorizing images into specific classes, making it a fundamental tool in various computer vision applications^23–26^. The underlying principle of CNNs lies in their ability to leverage 2-D convolution operations, which allow them to extract meaningful information from images. These convolution layers act as feature extractors, capturing important patterns, textures, and structures present in the input images.

In recent years, the field of computer vision has witnessed remarkable advancements, and numerous Convolutional Neural Network (CNN) architectures have emerged, each aiming to achieve higher accuracy on large benchmark datasets^27^. Given the context of limited data availability and the preference for smaller models due to their efficiency in training and inference, our selection of CNN architectures was strategic. Models like EfficientNet, MobileNetV3 (Large and Small), and ResNet18 are known for their compact architecture and computational efficiency, making them ideal for scenarios where data is scarce. Furthermore, these models lend themselves well to transfer learning approaches by leveraging pre-trained weights from these models, which were originally trained on large datasets, we can fine-tune them on our specific, smaller dataset to achieve significant improvements in performance. This strategy harnesses the learned features from vast, diverse datasets and applies them to our niche task of classifying tooth development stages. Among these architectures, ResNet is a standout architecture known for effectively addressing the vanishing gradient problem in deep CNNs. By introducing residual connections, information flows directly through the network, enabling ResNet to be deeper, more expressive, and achieve improved image classification performance. Another significant advancement in the realm of CNN architectures is MobileNet^28^. MobileNet models are lightweight and resource-efficient, perfect for deployment on less powerful devices like mobile phones and embedded systems. They utilize depthwise separable convolutions, which significantly reduce parameters and computations compared with traditional convolutions, resulting in faster inference times and optimal performance for real-time applications on resource-constrained devices. Similar to MobileNet models, ShuffleNet is a convolutional neural network specifically designed for mobile devices with limited computing power. This architecture employs two novel operations, namely pointwise group convolution and channel shuffle, to reduce computational costs while preserving accuracy.

EfficientNet is a groundbreaking CNN architecture that combines the strengths of ResNet and MobileNet while addressing their limitations^29^. It introduces a compound scaling method that optimizes the depth, width, and resolution of the network simultaneously using a single scaling coefficient. This allows users to find a well-balanced trade-off between model size and accuracy, making EfficientNet versatile and efficient for various image classification tasks. The architecture incorporates depth- wise separable convolutions from MobileNet and residual connections from ResNet, resulting in state-of-the-art performance on benchmark datasets with improved computational efficiency. In this study, we primarily use EfficientNet-B0 as our base model but we also compared it with various other publicly available CNN architectures. The general architecture of EfficientNet-B0 is given in Table 1.

**Table 1:** General structure of EfficientNetB0.

Layer	Output size	Details
Input layer	3 × 192 × 192	Input Image with 192 × 192 pixels
Convolutional layers	32 × 96 × 96	Standard Conv 3 × 3, Stride 2
Depthwise separable	32 × 96 × 96	Depthwise Conv 3 × 3, Pointwise Conv 1 × 1
Depthwise separable	16 × 96 × 96	Depthwise Conv 3 × 3, Pointwise Conv 1 × 1
Depthwise separable	24 × 48 × 48	Depthwise Conv 3 × 3, Pointwise Conv 1 × 1
Inverted residual	40 × 48 × 48	
Inverted residual	80 × 24 × 24	
Depthwise separable	80 × 24 × 24	Depthwise Conv 3 × 3, Pointwise Conv 1 × 1
Depthwise separable	112 × 12 × 12	Depthwise Conv 3 × 3, Pointwise Conv 1 × 1
Inverted residual	192 × 12 × 12	
Depthwise separable	192 × 12 × 12	Depthwise Conv 3 × 3, Pointwise Conv 1 × 1
Depthwise separable	320 × 6 × 6	Depthwise Conv 3 × 3, Pointwise Conv 1 × 1
Max pooling	320 × 2 × 2	Max Pooling 3 × 3, Stride 2
Fully connected	1280	
Fully connected	8	Final output with softmax activation

**Figure 3:**
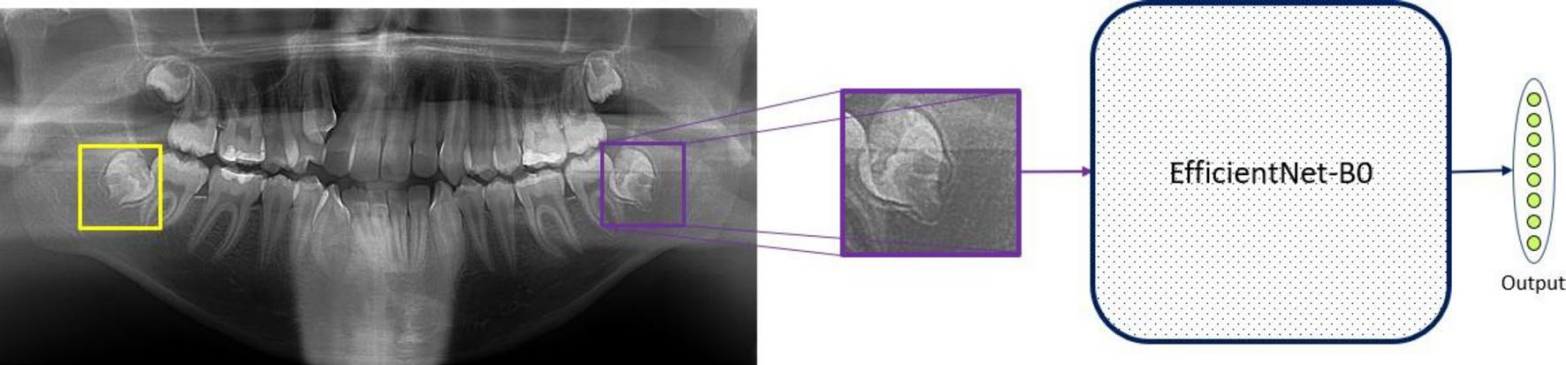
The model pipeline.

The model pipeline employed in our study is illustrated in Fig. 3. As a first step, we extract the Region of Interest (RoI) and remove any irrelevant parts from the panoramic X-ray images. By focusing only on the wisdom teeth region, we ensure that the model processes and classifies the most relevant information.

Once the RoI is extracted, the cropped images undergo data augmentation. This technique involves generating new samples by applying various transformations, such as rotation, auto contrast, and translation, to diversify the training data. Data augmentation helps to mitigate overfitting and improves the model’s ability to generalize to unseen images.Once the RoI is extracted, the segmented images undergo data augmentation. This technique involves generating new samples by applying various transformations, such as minor angular rotation, auto contrast adjustment, and small translations, to diversify the training data. Data augmentation helps to mitigate overfitting and improves the model’s ability to generalize to unseen images. The data augmentation applied involves three methods to enhance model generalization: slight random rotations (up to ± 10 degrees) to mimic orientation variability, automatic contrast adjustments (10% chance) to emphasize image features, and affine transformations that include translations (up to 10% of image dimensions) to simulate subject positioning variations. The augmented images are then fed into the model for classification. The model produces 8 outputs, representing the class probabilities of the input image belonging to each corresponding class. These probabilities indicate the confidence level with which the model assigns each image to a specific Demirjian stage, ranging from stage 3 to stage 10.

By utilizing this pipeline, our model effectively processes panoramic X-ray images, focuses on the wisdom teeth region, augments the data for better training, and provides accurate and confident predictions for the classification of the wisdom teeth into different developmental stages.

In the realm of medical applications, classification tasks often benefit from data-driven machine learning solutions. In these scenarios, datasets are meticulously constructed through rigorous statistical analysis of medical images. While simpler algorithms can sometimes yield satisfactory results, the superiority of CNN-based models becomes evident both in terms of performance and their capacity to reduce the need for manual intervention. These Convolutional Neural Network models, with their ability to automatically learn intricate features from the data, stand as a promising alternative, particularly in the medical domain, where precision and efficiency are paramount. By leveraging CNNs, we can enhance the accuracy and reliability of classifications while significantly minimizing the manual effort required.

### Focal loss

A loss function known as focal loss for handling imbalanced data was experimented with in this study^30^. First, it calculates the entropy loss (cross-entropy loss), between the model’s predictions (input) and the true labels (target). Then it computes the probability of classification (pt) by exponentiating the negated entropy loss. Finally, it determines the loss by applying a modulating factor to the entropy loss based on the focal loss formula.

The hyperparameter *γ* in the focal loss formula is a tunable parameter that controls the balance between easy and hard examples during training. By the grid search approach, a value of *γ* = 2 yielded the best results in our experiments.
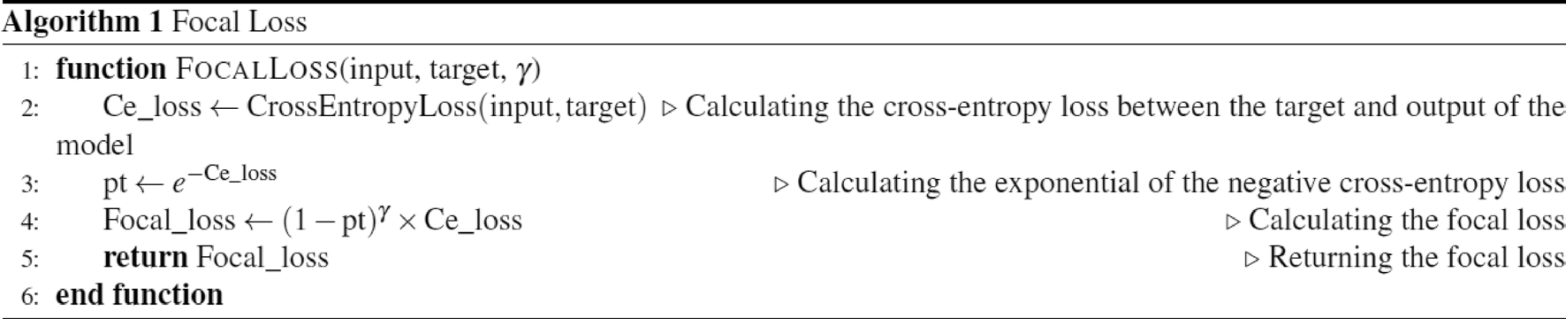


### The low-pass and high-pass pre-filters

We apply a low-pass and a high-pass filter as the initial filters of our neural network structure similar to a two-channel wavelet filterbank^31^.

A Gaussian filter is a low-pass filter that smoothens an image by reducing noise and blurring unnecessary details. It is helpful for reducing noise and irrelevant features. We apply the Gaussian filter to the original 2D image and generate a new image with a focus on important features.

On the other hand, a high-pass filter complements the low-pass filter by emphasizing the high-frequency components in an image, which correspond to edges and fine details. We also apply the high-pass filter to the original 2D image and generate a new image with a focus on edges and fine details. Combining these filterbank generated images with the original 2D images adds depth to the input to the neural network. With this method, the model incorporates characteristics to obtain an understanding of the input data. This can boost the capacity of the model to identify patterns and important specifics ultimately leading to outcomes, in tasks such, as recognizing objects or classifying them. The filter parameters are listed in Table 2.

**Table 2:** Descrptive statistics and independent student t-tests results (age in years)—gender and maturation stages for quadrant 3 Summary.

Stages	N	Mean	Female		N	Mean	Male		*p* value*
			SD	95% CI (LB-UB)			SD	95% CI (LB-UB)	
1-Missing Tooth	59	7.69	0.464	7.57–7.82	48	7.73	0.494	7.59–7.87	0.713
2-Empty Follicle	27	8.81	1.039	8.40–9.23	13	9.23	1.536	8.30–10.16	0.318
3-A	115	9.38	1.031	9.19–9.57	117	9.31	0.782	9.16–9.45	0.533
4-B	176	9.95	1.507	9.73–10.18	191	9.72	1.299	9.53–9.90	0.106
5-C	312	11.87	2.168	11.63–12.11	296	11.36	2.210	11.11–11.62	0.005*
6-D	311	13.31	1.505	13.14–13.48	221	13.30	1.447	13.11–13.50	0.996
7-E	236	14.13	1.846	13.89–14.37	198	13.95	0.914	13.83–14.08	0.220
8-F	150	15.76	2.882	15.30–16.22	155	15.46	2.579	15.06–15.87	0.346
9-G	111	18.50	3.675	17.81–19.20	98	17.49	3.240	16.84–18.14	0.035*
10-H	344	24.06	1.556	23.89–24.22	217	23.79	2.108	23.51–24.07	0.110

To utilize both the low and high frequency components of an image, we employ six filters, two of which pass low frequencies while the rest serve as high-pass filters. The use of multiple filters is crucial as images contain essential information that may reside in different orientations and frequency ranges. Two of the high-pass filters are known as Sobel operators, which are often employed as edge detectors for identifying edges in both vertical and horizontal directions. The original images and the resulting outputs of each filter are shown in the Appendix.

**Table:** 

Filter type	Filter matrix	Application
Low-pass filters	$${{\left[ {\begin{array}{*{20}l} 1 \hfill & 2 \hfill & 1 \hfill \\ 2 \hfill & 4 \hfill & 2 \hfill \\ 1 \hfill & 2 \hfill & 1 \hfill \\ \end{array} } \right]} \mathord{\left/ {\vphantom {{\left[ {\begin{array}{*{20}l} 1 \hfill & 2 \hfill & 1 \hfill \\ 2 \hfill & 4 \hfill & 2 \hfill \\ 1 \hfill & 2 \hfill & 1 \hfill \\ \end{array} } \right]} {16.0}}} \right. \kern-0pt} {16.0}}\quad {{\left[ {\begin{array}{*{20}c} 1 & 4 & 6 & 4 & 1 \\ 4 & {16} & {24} & {16} & 4 \\ 6 & {24} & {64} & {24} & 6 \\ 4 & {16} & {24} & {16} & 4 \\ 1 & 4 & 6 & 4 & 1 \\ \end{array} } \right]} \mathord{\left/ {\vphantom {{\left[ {\begin{array}{*{20}c} 1 & 4 & 6 & 4 & 1 \\ 4 & {16} & {24} & {16} & 4 \\ 6 & {24} & {64} & {24} & 6 \\ 4 & {16} & {24} & {16} & 4 \\ 1 & 4 & 6 & 4 & 1 \\ \end{array} } \right]} {256.0}}} \right. \kern-0pt} {256.0}}$$	Applied to original 2D images for noise reduction and blurring of irrelevant details
High-pass filters	$$\left[ {\begin{array}{*{20}c} { - 1} & { - 1} & { - 1} \\ { - 1} & 8 & { - 1} \\ { - 1} & { - 1} & { - 1} \\ \end{array} } \right]\quad \left[ {\begin{array}{*{20}c} 0 & { - 1} & 0 \\ { - 1} & 4 & { - 1} \\ 0 & { - 1} & 0 \\ \end{array} } \right]$$	Emphasizes edges and fine details in original 2D images
Sobel operator (Ver.)/(Hor.)	$$\left[ {\begin{array}{*{20}c} { - 1} & { - 2} & { - 1} \\ 0 & 0 & 0 \\ 1 & 2 & 1 \\ \end{array} } \right]\quad \left[ {\begin{array}{*{20}c} { - 1} & 0 & 1 \\ { - 2} & 0 & 2 \\ { - 1} & 0 & 1 \\ \end{array} } \right]$$	Highlights horizontal and vertical edges

### Ethics approval and consent to participate

In this research publication, we conducted a retrospective study utilizing archived data. All samples used in the study was deidentified to ensure confidentiality. “Informed consent was waived by The University of Illinois Chicago’s (UIC)Office for the Protection of Research Subjects (OPRS) Institutional Review Board (IRB)” providing IRB-exempt status to the study with the assigned Study ID: (2021-0480). All experimental protocols were approved by The University of Illinois Chicago’s IRP and OPRS

## Results

**Figure 4:**
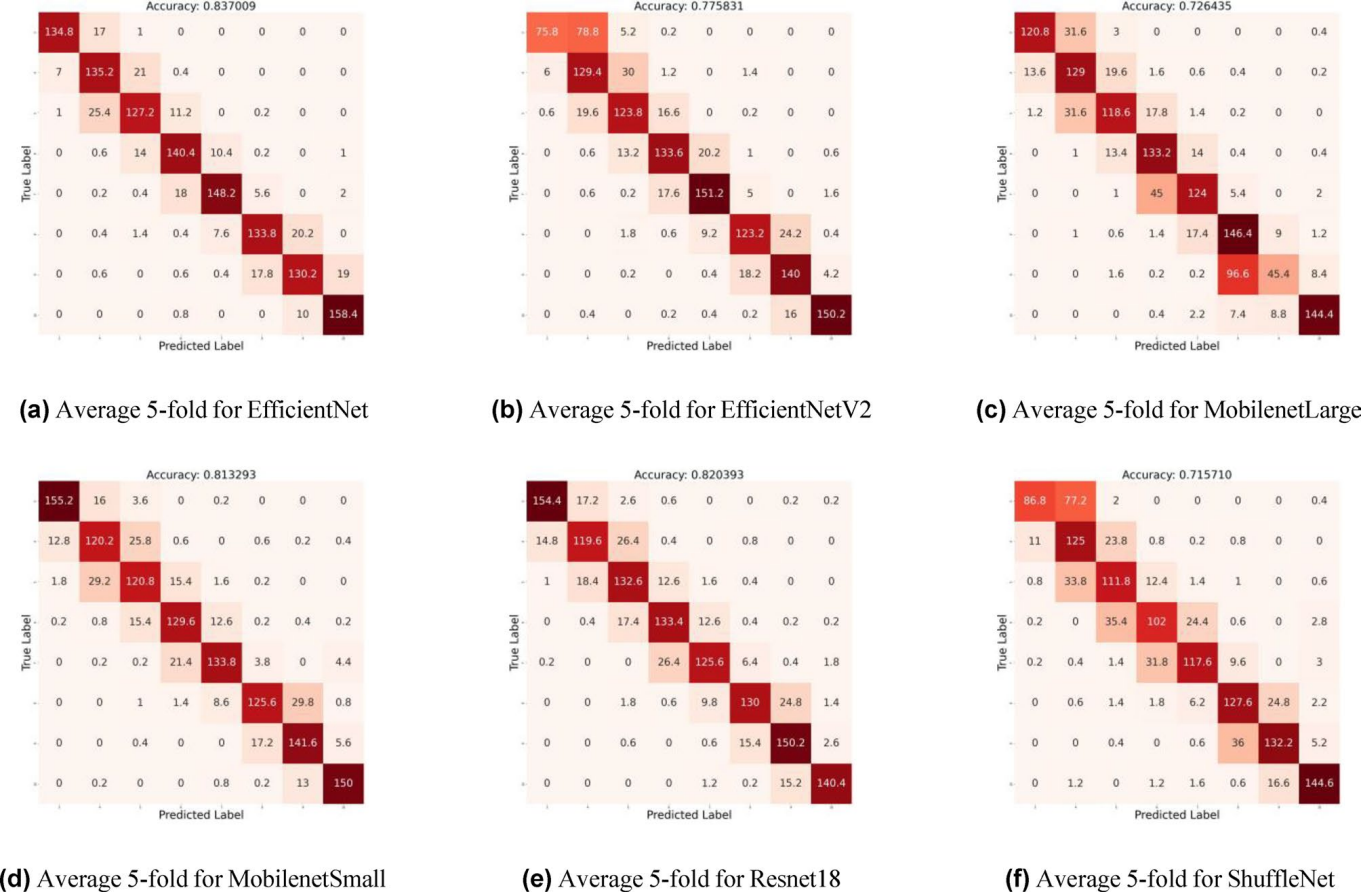
Illustration of the average fivefold confusion matrix.

Descriptive statistics and Independent Student t-test were used to evaluate the variables of age by sex and the stages of quadrants 3 and 4. Statistical significance was set at 0.05. The statistical data analysis was performed using Software IBM SPSS Statistics for Widows, version 29.0 (IBM Corp., Armonk, N.Y., USA^32^. A summary of our key findings includes detailed accuracy percentages and significant observations which have emerged from our extensive analysis, notably highlighted in Tables 4, 5 and Figs. 4, 8. Our investigation uncovers significant variations in the performance of various model architectures, which highlights the complexity of selecting the optimal model for dental diagnostics. EfficientNet stands out for its superior accuracy, a testament to its balanced scaling of depth, width, and resolution, which likely contributes to its enhanced ability to handle the intricate details found in dental imagery. ResNet18, though slightly less accurate than EfficientNet, still demonstrates robust performance, possibly due to its deep residual learning framework that aids in learning from a vast array of dental images without succumbing to the vanishing gradient problem. MobileNetV3, on the other hand, offers competitive accuracy under certain conditions, benefiting from its lightweight structure and efficiency, making it particularly suitable for scenarios requiring quick analysis or deployment on mobile devices. The distinct performance metrics of these models under various conditions suggest a nuanced landscape of model applicability, where the choice of model architecture could be tailored to specific requirements of accuracy, efficiency, and computational resource availability in dental diagnostics. The overall sample mean age was 14.47 years (SD = 5.20) with a median of 14.00 years. The sample was composed of 54.3% females and 45.7% males. The mode of the stages in quadrants Q3 and Q4 is 5. The total mean age of Q3 and Q4 ranged from 8 to 24 years, for both males and females, across all 10 maturation stages. The mean age of the females (14.88 years) was statistically significantly higher than that of males (14.00 years) with a mean difference of 0.866 years (*p* value < 0.001). In Q3 the mean age of females (14.88 years) was also significantly higher than that of males (14.01 years), with a mean difference of 0.880 years, (*p* value < 0.001). There was a statistically significant mean difference on age between males and females for stages 5 and 9, with *p* values of 0.005 and 0.035, respectively. In Q4, the mean age of females (14.91 years) was also significantly higher than that of males (14.02 years), with a mean difference of 0.886 years, (*p* value < 0.001). There was a statistically significant mean difference on age between males and females for stages 2, 5 and 9 with *p* values 0.045, 0.019 and 0.007, respectively Tables 2 and 3. Having established the statistical significance of age and gender differences in the stages of quadrants 3 and 4 using the IBM SPSS Statistics Software, version 29.0, the study’s focus shifts towards leveraging advanced computational techniques. This transition marks a pivotal point in our research, where traditional statistical methods meet the cutting-edge realm of machine learning.

**Table 3:** Descrptive statistics and independent student t-tests results (age in years)—gender and maturation stages for quadrant 4 Summary.

Stages	N	Mean	Female		N	Mean	Male		*p* value*
			SD	95% CI (LB-UB)			SD	95% CI (LB-UB)	
1- Missing Tooth	56	7.7	0.464	7.57–7.82	48	7.73	0.494	7.59–7.87	0.728
2- Empty Follicle	25	8.6	0.764	8.28–8.92	22	9.09	0.868	8.71–9.48	0.045*
3-A	117	9.44	1.037	9.25–9.63	121	9.34	0.900	9.5–9.22	0.441
4-B	166	10.04	1.572	9.80–10.28	180	9.82	1.419	9.61–10.03	0.184
5-C	324	11.86	2.180	12.10–11.85	296	11.40	2.235	11.19–11.70	0.019*
6-D	310	13.31	1.450	13.14–13.47	216	13.23	1.537	13.02–13.43	0.546
7-E	238	14.16	1.901	13.92–14.41	199	13.96	0.828	13.84–14.08	0.136
8-F	146	15.62	2.712	15.18–16.07	149	15.52	2.655	15.09–15.95	0.750
9-G	111	18.54	3.664	17.84–19.24	96	17.25	3.023	16.64–17.86	0.007*
10-H	369	24.02	1.680	23.84–24.19	222	23.71	2.273	23.41–24.01	0.085

We also implement a k-fold cross-validation technique to validate the effectiveness of our study. This technique involves dividing the dataset into k subsets (folds), with each fold serving as both a training and testing set in rotation. By repeating this process k times, we obtain a comprehensive evaluation of our proposed methodology on different subsets of the data. K-fold cross-validation is used to ensure the model’s generalizability to diverse datasets. This widely used validation technique assesses whether the model learns the image context or merely memorizes specific examples. Unlike traditional training and testing data separation, k-fold cross-validation dynamically determines training and testing sets in each fold, making the evaluation more robust. We adopt *k* = 5 in our experiments.

For the 5-fold cross-validation, a training set comprising 5300 images is used. The split ratio of 4:1 ensures non-overlapping between the training and validation folds. Prior to augmentation, images are divided into the respective folds, and the augmentation process is then applied independently. This approach guarantees that each training and validation fold remains distinct throughout the training process. The iterative process of selecting the model components shapes the final structure of our model, ensuring its capability to accurately classify wisdom teeth across diverse data sets.

In this section, a comparison of various Convolutional Neural network (CNN) models is presented. The models are trained and their performance evaluated using the dataset through a rigorous 5-fold cross-validation process. For each fold, the accuracy metric is calculated, and the results are shared in the table. For a comprehensive comparison, different learning rates and loss functions are considered. As the optimizer, we are using AdamW with the scheduler, which gradually decreases the learning rate for better convergence and finding the optimal solution. Various comparisons are presented with different sets of hyperparameters. To enhance the clarity of the classification results, we calculate the average confusion matrix to evaluate its performance.

Table 4 compares the performance of various machine learning models under specific conditions, namely with a learning rate of 0.001 and employing the Focal loss function. Each row represents a different model: EfficientNet, EfficientNetV2, MobileNetV3Large, MobileNetV3Small, Resnet18, and ShuffleNet. The table presents the performance of each model with and without the pre-filtering. Across the different folds, the models exhibited varying levels of effectiveness. To gain a precise and accurate assessment of the models’ performance, we calculated the mean of the accuracy results obtained across the five folds. Notably in Table 4, EfficientNet with the pre-filtering achieved the highest performance, boasting a mean accuracy of 83.76%, while ShuffleNet without the pre-filtering performed relatively lower, reaching a mean accuracy of 75.21%. EfficientNet’s remarkable performance was followed by ResNet18 with pre-filtering, achieving an average accuracy of 81.95%.

**Table 4:** Performance comparison of models trained with 0.001 learning rate and by using focal loss.

Model	Prefilter applied	Prefilter not applied
EfficientNet	%83.17	%83.76
EfficientNetV2	%81.34	%80.57
MobileNetV3Large	%78.69	%77.75
MobileNetV3Small	%81.39	%78.85
Resnet18	%81.47	%81.95
ShuffleNet	%75.21	%70.44

EfficientNet achieved the highest mean accuracy across the folds in this specific experimental setup. Other models tested, including EfficientNetV2, MobileNetV3Small, and ResNet18, also demonstrated competitive performance. Detailed performance for each model is provided in Table 4.

To further evaluate the models’ performance, we conducted experiments using the cross-entropy loss function. Similar to previous trials, we employed learning rate of 0.001 to train the models with the cross-entropy cost function. Table 5 presents a comparative analysis of the models’ performance under these conditions. Specifically, Table 5 showcases the mean of the accuracy levels across the 5 folds of models trained with a learning rate of 0.001 and the cross-entropy loss function. As in the previous tables, the evaluation was conducted using a cross-validation approach, where the dataset was divided into different subsets or “folds” for training and testing. In these experiments, we display the performance of the model with the existence of the preprocessing filters to show their impact. Analyzing the results, we can see that the models exhibit varying levels of performance across different folds. Similar to previous experiments, EfficientNet with the filters achieves the highest mean accuracy at 83.35%, while ShuffleNet with no filters performs lower at 75.17%. The standard deviation of the accuracy levels of EfficientNet with filters provided is as low as 1.03%, indicating a sustainable performance. Under these specific conditions of a learning rate of 0.001 and using cross-entropy loss, EfficientNet with preprocessing filters again emerges as the top-performing model, demonstrating consistent and robust performance across different folds. Resnet18 and MobileNetV3Small with filters also show competitive performance with an average accuracies of 81.71% and 80.74%. However, EfficientNetV2, MobileNetV3Large, and ShuffleNet exhibit comparatively lower average accuracies. It showed the impact of preprocessing filters in these settings. The application of filters leads to notable performance enhancements in EfficientNet and ResNet18 models.

**Table 5:** Performance comparison of models trained with 0.001 learning rate and by using cross entropy loss.

Model	Prefilter applied	Prefilter not applied
EfficientNet	%83.07	%83.35
EfficientNetV2	%78.91	%78.76
MobileNetV3Large	%79.82	%77.45
MobileNetV3Small	%81.57	%80.74
Resnet18	%80.29	%81.71
ShuffleNet	%75.17	%69.44

A higher learning rate resulted in divergence in our experiments with models. Given our small dataset, we observed that smaller models, such as EfficientNet-B0 and MobileNetV3Small, outperformed larger ones like EfficientNetV2 and MobileNetV3Large.

Throughout the experiments conducted in this study, EfficientNet with pre-filtering applied consistently emerged as the top performer, demonstrating reliable and robust performance across different folds and experimental setups. Its more sophisticated counterpart, EfficientNetV2, delivered a commendable performance but fell short by a narrow margin. ResNet18 consistently achieved the second-highest performance, indicating that the task is well-suited for conventional convolutional neural networks (CNN) as it consistently attained an average of around 80% accuracy. MobileNetV3Large proved to be the worst performer among the compared models, often diverging as the learning rate increased. The experiments also reinforced the significance of incorporating preprocessing filters. The pinnacle performance across all experiments was the mean accuracy level of 83.76% achieved by EfficientNet with filters when trained with the Focal loss and a learning rate of 0.001. As a final observation regarding model performance, the introduction of preprocessing filters consistently reduced the mean accuracy of ShuffleNet.

We attribute this to the inherent nature of ShuffleNet, which randomly shuffles channels after pointwise group convolutions. Since the filters generate both high and low components along with the original image and feed them into the model, the shuffling may hinder its performance. We also show the average confusion matrices of all the models in Fig. 4. Given our small dataset, we observed that smaller models, such as EfficientNet-B0 and MobileNetV3Small, outperformed larger ones like EfficientNetV2 and MobileNetV3Large. Additionally, we noticed that as the learning rate increased, the training process did not converge properly.

In summary, among the architectures tested, EfficientNet demonstrated superior performance, achieving a classification accuracy of 83.7%. Other architectures also showed promising results, with accuracies ranging from 71.57 to 82.03%. These outcomes indicate the effectiveness of our proposed methodology in accurately classifying the developmental stages of lower wisdom teeth in OPG images.

## Discussion

Determination of tooth development stages and age estimate through radiographic evaluation is of great importance in the medical, dental and forensic fields as it is used to enhance diagnostic accuracy and improve identification of individuals. Several methods have been used to determine the most accurate way to estimate dental age. Demirjian et al.^33^, Gleiser and Hunt^34^, Moorrees et al.^35^, Gustafson and Koch^36^, Harris and Nortje^37^, Kullman et al.^38^, proposed methods that were tested in the past. In 2004, Olze et al. studied those methods, while concluding that the most accurate results were obtained with Demirjian et al. classification system due to providing a sufficient number of stages as well as defining them in lieu of length estimations. Demirjian et al. classification showed the highest values for both observer agreement and for correlation between the stages as defined by the method and true age^39^. Demirjian presented a classification method differentiating eight stages of crown and root development, allowing for the evaluator to define stages by changes in shape with no metric estimations needed. Therefore, making it more objective and simpler^7,33–40^.

Implementing an automated tool is highly beneficial. It reduces human errors, ensuring more reliable results, and quickly determines dental age, aiding identification. Introducing an accurate automated tool for dental age assessment based on the Demirjian classification system overcomes human interpretation issues, speeding up the process and enhancing precision, especially in legal and forensic contexts. Overall, this article highlights an innovative approach to estimating dental age using panoramic radiographs and offers insights into the potential benefits and challenges of automating the process. Reflecting on our initial research objectives and hypotheses, the application of convolutional neural networks (CNNs) for estimating the developmental stages of lower third molars has demonstrated a notable alignment with our expectations. Our hypothesis posited that advanced deep learning techniques could significantly enhance the accuracy and reliability of dental age estimation, a proposition that our results robustly support. We collected and analyzed dental images and data and used deep learning, like convolutional neural networks (CNNs), to extract key information from these images. This information includes details about tooth structure and mineralization patterns.Our comparative analysis of various CNN architectures revealed distinct performance characteristics, with EfficientNet outperforming others in accuracy. This variation can be attributed to EfficientNet’s scalable architecture, which optimizes depth, width, and resolution more effectively for image classification tasks. Conversely, the lower performance of architectures like MobileNetV3Large underlines the challenges of balancing model complexity with the specificity of dental radiographic images.

There are some studies used AI to determine mandibular third molar position and impaction status. Maruta et al.^41^ made a significant contribution to the diagnosis of impacted mandibular third molar classification by developing an automated machine learning-based system that effectively utilizes both the Pell and Gregory and Winter classifications as annotations in their image data. Their work demonstrated the potential of machine learning in accurately identifying the impacted status of mandibular third molars, a critical aspect of dental diagnosis and treatment planning. Building upon Maruta et al. work, Sukegawa et al. conducted a comprehensive evaluation of a multi-task learning approach for classifying mandibular third molar teeth position^42^. Their study explored the effectiveness of this approach in simultaneously predicting the impacted status and the position of the mandibular third molar. The results of their evaluation highlighted the advantages of multi-task learning in improving classification accuracy and providing additional insights into the anatomical characteristics of mandibular third molars. Further advancing the field of impacted mandibular third molar analysis, Celebi delved into the segmentation and detection of these teeth^43^. Celebi proposed a precise segmentation method using a YOLO-type network, demonstrating its capability to accurately identify the boundaries of impacted mandibular third molars. This work holds promise for developing automated segmentation tools that can aid in dental diagnosis and treatment planning. However, these articles did not attempt to classify the third molar development stages. In the literature there is one research group who made attempts to classify the lower third molar development stages. De Tobel et al.^20^ study focused on automating the staging of lower third molar development in panoramic radiographs. They used a dataset comprising twenty radiographs per developmental stage, categorized by gender. The methodology involved Deep Learning Convolutional Neural Networks (CNNs) for stage recognition, and their findings showed the effectiveness of this approach in dental age estimation^20^. Another study focused on staging third molar development in panoramic radiographs, 400 images were analyzed using an AlexNet Deep Convolutional Neural Network (CNN) and various segmentation methods. The study tested bounding box, rough, and full tooth segmentation techniques to determine their impact on stage allocation accuracy. Full tooth segmentation showed the most promising results in improving the accuracy of automated dental stage allocation for age estimation^44^. Also, another study explored automated age estimation using 400 panoramic radiographs, focusing on third molar development. It compared different segmentation methods and found full tooth segmentation with a DenseNet201 CNN to be most accurate for stage allocation, enhancing the precision of dental age estimation. The study focused on fully automating the staging of third molar development using convolutional neural networks (CNNs). Employing a dataset of 400 panoramic radiographs, with 20 images per developmental stage per sex, the study employed a three-step CNN process for localization, segmentation, and classification of the third molar^45^.

Unlike De Tobel et al.^20^ research and other studies, which utilized a dataset of twenty radiographs or 400 panoramic radiographs per developmental stage and employed Deep Learning Convolutional Neural Networks (CNNs) for stage recognition, our study introduces a more comprehensive approach. We combine Region of Interest (ROI) extraction with high-pass and low-pass filters, alongside extensive data augmentation techniques like rotation, translation, and noise introduction. This multifaceted approach not only refines image preprocessing for improved classification accuracy but also significantly enhances the model’s ability to generalize and maintain robustness against unseen data points. Despite the promising results achieved, this study has limitations that warrant consideration. The potential impact of imbalanced data on our models’ learning efficacy underscores the need for further investigation with a more balanced dataset. Moreover, while the study focuses on the develop- mental stages of lower third molars, correlating these stages to chronological age across different genders and ethnic groups remains an area for future research. Future research should aim to include a more balanced dataset.

When correlating the lower third molar development stages to the corresponding chronological age, our data showed that same stages showed differences between males and females. Our finding agrees with previous studies by Olze et al.^46^, and Sisman et al.^47^. In their study third molars developed earlier in males than females showing a strong correlation between age and third molar development for both sexes supporting the data presented in this study. However in our study our model focused only on the classification of the lower third molar developmental stages. Future studies are needed to correlate the developmental stages with the estimated chronological age in different genders and races^46,47^. Comparing our findings with existing literature, particularly the studies by Olze et al. and others, underscores the advancements our research contributes to the field of dental age estimation. By leveraging a comprehensive approach that combines ROI extraction, filtering techniques, and CNNs, our study not only confirms the efficacy of the Demirjian classification system but also extends its application through automation, offering a novel pathway for enhancing diagnostic precision in dental radiology. Looking forward, our study opens several avenues for future research, including exploring the integration of alternative deep learning models, extending our methodology to broader datasets encompassing diverse ethnic backgrounds, and examining the longitudinal reliability of automated dental age estimation. Such investigations could further refine the accuracy and applicability of AI in dental diagnostics.

This retrospective study introduces a novel machine-learning-based approach to accurately estimate the developmental stages of lower third molars in orthopantomograms (OPG) images. By leveraging the power of convolutional neural networks (CNNs), we have developed a robust classification model that can effectively process input OPG images and classify them into their corresponding developmental stages. This methodology has the potential to revolutionize the field of dental radiology by providing a more efficient and accurate method for assessing lower third molar development. The proposed CNN classification model incorporates two preprocessing filters, a high-pass filter and a low-pass filter, which play crucial roles in enhancing the accuracy of the classification process. The high-pass filter effectively extracts essential edge information from the OPG image, while the low-pass filter efficiently eliminates image noise, ensuring that the model focuses on the relevant features. The combination of these preprocessing filters with the EfficientNet architecture, a renowned CNN architecture for image classification tasks, enables the model to achieve superior performance in estimating lower third molar developmental stages. It is important to note that while an automated accurate tool can offer numerous benefits, it should not entirely replace human expertise. The tool should be seen as a valuable adjunct to assist dental professionals and other relevant experts in their assessments. The collaboration between automation and human expertise can result in more precise and reliable dental age estimation. AI extends its utility by furnishing dentists with evidence-based recommendations and treatment insights. These recommendations draw from vast datasets and expert knowledge, facilitating treatment planning and enhancing clinical decision-making. AI-powered systems assume the role of virtual mentors, delivering feedback and guidance to dentists throughout their training. By evaluating dentists’ OPG image interpretations, AI supports skill refinement and improved accuracy over time. AI acts as a dependable second opinion by cross-referencing its analysis with that of a dentist, potentially detecting overlooked details or providing alternative viewpoints. AI techniques are also proficient at enhancing the quality of OPG images^48^. It is crucial to recognize that AI does not aim to replace human dentists but rather to complement their expertise and offer supplementary assistance. By harnessing the combined strengths of AI and dental professionals, we can elevate the precision, efficiency, and quality of OPG evaluations. This research introduces a novel and powerful approach to the automated classification of tooth development stages, contributing significantly to the fields of dental diagnostics and treatment planning.

By demonstrating the potential of advanced machine learning models and a multifaceted methodological approach, our findings suggest a transformative direction for improving dental and forensic analysis techniques.

## Conclusion

This study introduces a machine learning framework that accurately categorizes the developmental stages of lower third molars in orthopantomograms (OPG). The method achieves an accuracy rate of 83.7% by employing a novel convolutional neural network (CNN) which integrates two trainable preprocessing filters into the pre-trained EfficientNet architecture. Our findings indicate that EfficientNet achieved the highest classification accuracy at 83.7%. Other architectures achieved accuracy ranging from 71.57 to 82.03%. These filters, a high-pass filter for edge enhancement and a low-pass filter for noise reduction enhance the clarity and relevance of the imaging data processed by the model. This contribution advances dental radiology by furnishing a dependable tool for dental age estimation and forensic analysis. Studies can look into the proposed model’s applicability to additional dental imaging tasks and evaluate its performance across diverse patient demographics.

### Supplementary Information

Supplementary Information.

## Data Availability

All data generated during this study are included in this published article and its supplementary information files. The data analyzed in this study are available from The University of Illinois Chicago but restrictions apply to the availability of these data, which were used under license for the current study, and so are not publicly available.
