# Author Correction: A fully automated classification of third molar development stages using deep learning

**DOI:** 10.1038/s41598-024-66731-5

**Published:** 2024-07-10

**Authors:** Omid Halimi Milani, Salih Furkan Atici, Veerasathpurush Allareddy, Vinitha Ramachandran, Rashid Ansari, Ahmet Enis Cetin, Mohammed H. Elnagar

**Affiliations:** 1 Department of Electrical and Computer Engineering, University of Illinois Chicago, Chicago, IL USA; 2 Department of Orthodontics (M/C 841), College of Dentistry, University of Illinois Chicago, 801 S. Paulina Street, RM 131, Chicago, IL 60612-7211 USA

Correction to: *Scientific Reports* 10.1038/s41598-024-63744-y, published online 07 June 2024

The original version of this Article contained an error in the name of Mohammed H. Elnagar, which was incorrectly given as Mohammad H. Elnagar.

The original Article has been corrected.

