# Supplementary material for: A fully automated classification of third molar development stages using deep learning
Source: Sci Rep. 2024 Jun 7;14:13082. doi: 10.1038/s41598-024-63744-y (PMC11156840; doi:10.1038/s41598-024-63744-y)
Supplement: Supplementary file 1 — Supplementary Information. [file 41598_2024_63744_MOESM1_ESM.pdf]

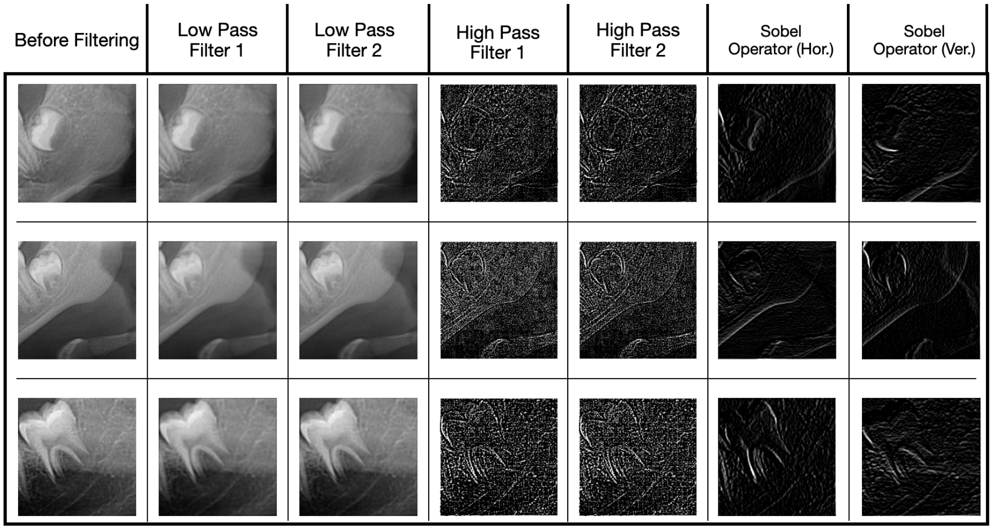

Figure 5. Illustration of the outcome after applying filters Gaussian and High-pass Filter to the original images.

Appendix

In the Appendix, we would like to present additional details about our study. To illustrate the outputs of the preprocessing filters, we have included a table in Figure 5. The table presents three image samples from three distinct classes on the leftmost column. In each subsequent column, the output of each filter is displayed. As you can observe, the filters effectively extract various features from the images, providing the model with enhanced information for improved classification.

As we previously stated, the preprocessing filters undergo training over the epochs, adapting their weights based on the gradient of the cost function. The final configuration of the filters is obtained and presented in Figure 6. The figure depicts the spatial arrangement of the filters, showcasing their varying shapes and magnitudes. This visual representation provides insights into the filters' adaptability.

Before Training	After Training	Before Training	After Training
**High Pass Filter 1** $\begin{bmatrix}-1 & -1 & -1\\-1 & 8 & -1\\-1 & -1 & -1\end{bmatrix}$	$\begin{bmatrix}-0.99 & -0.99 & -0.99\\-0.99 & 8.01 & -0.99\\-1 & -0.99 & -0.98\end{bmatrix}$	**Low Pass Filter 2** $\begin{bmatrix}0.0039 & 0.0156 & 0.0234 & 0.0156 & 0.0039\\0.0156 & 0.0625 & 0.0938 & 0.0625 & 0.0156\\0.0234 & 0.0938 & 0.1406 & 0.0938 & 0.0234\\0.0156 & 0.0625 & 0.0938 & 0.0625 & 0.0156\\0.0039 & 0.0156 & 0.0234 & 0.0156 & 0.0039\end{bmatrix}$	$\begin{bmatrix}0.0063 & 0.0196 & 0.0189 & 0.0097 & -0.0051\\0.0241 & 0.0680 & 0.0953 & 0.0635 & 0.0115\\0.0273 & 0.0927 & 0.1349 & 0.0918 & 0.0238\\0.0126 & 0.0578 & 0.0878 & 0.0592 & 0.0208\\-0.0006 & 0.0140 & 0.0242 & 0.0250 & 0.0190\end{bmatrix}$
**High Pass Filter2** $\begin{bmatrix}0 & -1 & 0\\-1 & 4 & -1\\0 & -1 & 0\end{bmatrix}$	$\begin{bmatrix}-0.01 & -1 & -0.01\\-1 & 3.98 & -1\\0 & -0.99 & -0.01\end{bmatrix}$	**Sobel Operator (Hor.)** $\begin{bmatrix}-1 & 0 & 1\\-2 & 0 & 2\\-1 & 0 & 1\end{bmatrix}$	$\begin{bmatrix}-1.05 & -0.04 & 0.95\\-2.05 & -0.07 & 1.93\\-1.04 & -0.05 & 0.92\end{bmatrix}$
**Low Pass Filter 1** $\begin{bmatrix}0.0625 & 0.125 & 0.0625\\0.125 & 0.25 & 0.125\\0.0625 & 0.125 & 0.0625\end{bmatrix}$	$\begin{bmatrix}0.11 & 0.15 & 0.09\\0.16 & 0.31 & 0.17\\0.08 & 0.16 & 0.13\end{bmatrix}$	**Sobel Operator (Ver.)** $\begin{bmatrix}-1 & -2 & -1\\0 & 0 & 0\\1 & 2 & 1\end{bmatrix}$	$\begin{bmatrix}-0.96 & -1.95 & -0.94\\0.04 & 0.05 & 0.04\\1.04 & 2.02 & 1.03\end{bmatrix}$

Figure 6. The filter coefficients before and after the training.

To illustrate the model's convergence behavior, we present the test accuracy and test loss plots in Figure 7. The models were trained with the preprocessing filters, as their effectiveness has been demonstrated in training under the default training settings.

Finally, we present the confusion matrices, this time with percentages to provide a clearer representation of the classification performance. The matrices were generated using the default training settings, specifically a learning rate of 0.001 and the Focal loss function. The matrices offer insights into the model's ability to correctly identify and classify images from different classes. The matrices are shown in Fig. 8

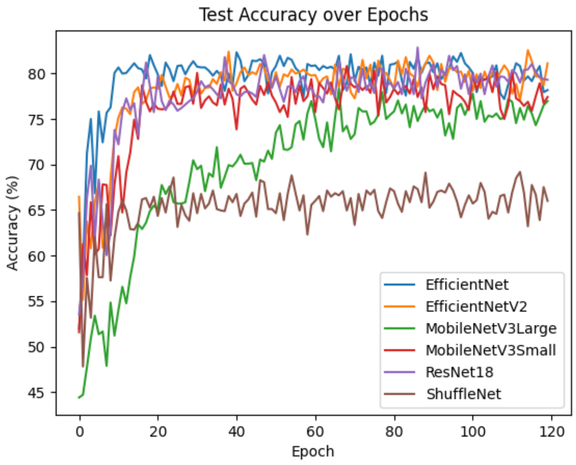

(a) Accuracy.

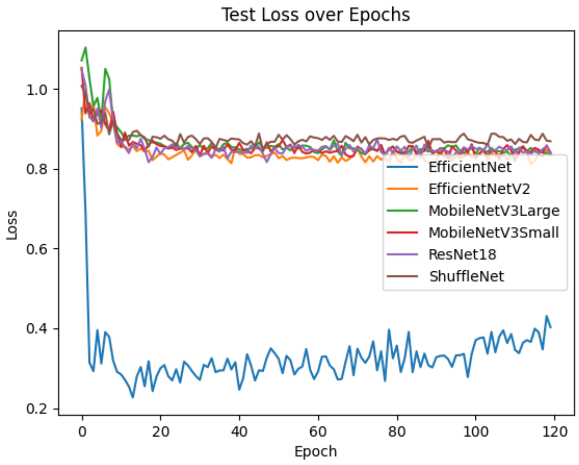

(b) Loss.

Figure 7. Test accuracy and test loss versus the number of epochs of different networks. The EfficientNet reaches the highest accuracy and converges to a minimum loss.

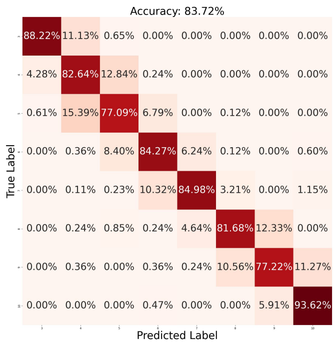

(a) Average 5-fold for EfficientNet

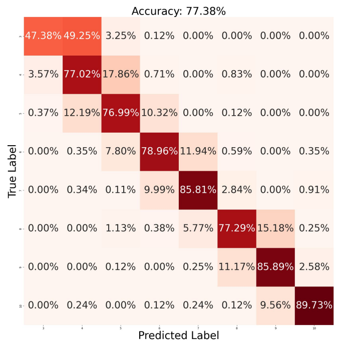

(b) Average 5-fold for EfficientNetV2

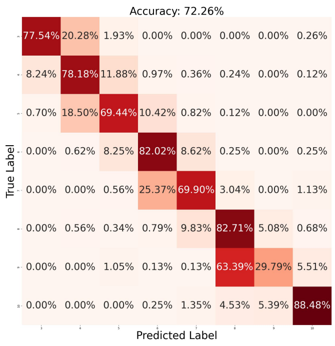

(c) Average 5-fold for MobilenetLarge

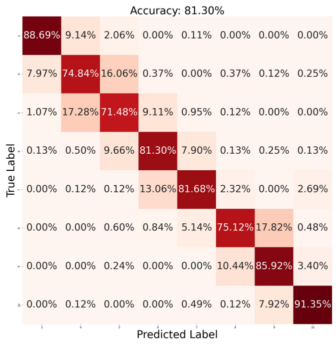

(d) Average 5-fold for MobilenetSmall

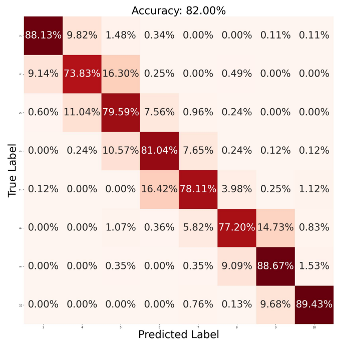

(e) Average 5-fold for Resnet18

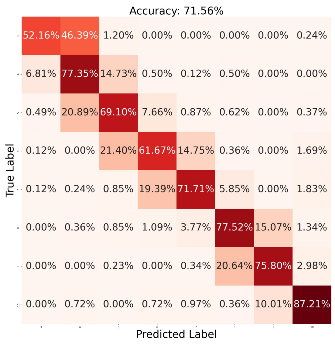

(f) Average 5-fold for ShuffleNet

Figure 8. Illustration of the percentage 5-fold confusion matrix.
